# Insulin treatment corrects hepcidin but not YKL-40 levels in persons with type 2 diabetes mellitus matched by body mass index, waist-to-height ratio, C-reactive protein and Creatinine

**DOI:** 10.1186/s12902-017-0204-4

**Published:** 2017-08-25

**Authors:** Driton Vela, Jovica Leshoski, Zana Vela, Muharrem Jakupaj, Mitko Mladenov, Ramadan B. Sopi

**Affiliations:** 1 Faculty of Medicine, University of Prishtina, Martyr’s Boulevard n.n, 10000 Prishtina, Kosovo; 2 Institute of Biology, Faculty of Natural Sciences, “Sts. Cyril and Methodius” University, Skopje, 1000 Macedonia

**Keywords:** Hepcidin, YKL-40, Diabetes

## Abstract

**Background:**

It has been shown that hepcidin and YKL-40 levels change in persons with insulin resistance in different circumstances. However, variations of the levels of these parameters through the stages of prediabetes and type 2 diabetes mellitus are unclear. We hypothesized that hepcidin levels will decrease in persons with prediabetes, while these levels will tend to correct when persons with diabetes are treated with insulin. Finally we sought to determine the levels of YKL-40 in all groups of participants included in the study.

**Methods:**

Serum hepcidin levels and YKL-40 levels were measured in control group (*n* = 20), persons with prediabetes (*n* = 30) and persons with diabetes on insulin therapy (*n* = 30) using ELISA method. Patients in all three groups were matched by Body Mass Index, Waist-to-Height Ratio, C-Reactive Protein and creatinine levels.

**Results:**

Hepcidin levels were lower in persons with prediabetes compared to control, while persons with diabetes on insulin therapy had higher values than those with prediabetes (*p* = 0,00001). YKL-40 levels showed no significant changes.

**Conclusions:**

Serum hepcidin levels in matched persons with prediabetes are a stronger marker of early changes in glucose metabolism compared to YKL-40 levels. Also, treatment with insulin corrects hepcidin levels, but not YKL-40 levels. Correcting levels of hepcidin is important for reducing iron-overload, which is a risk factor for diabetes.

## Background

Since its discovery as a major regulator of iron metabolism, hepcidin has become a focal point of studies through which scientists are trying to understand the role of this protein in different circumstances and pathologies. Hepcidin is a protein composed of 25 aminoacids produced in hepatocytes, but also in macrophages, adipocytes, tubular cells of the kidneys [1]. After being secreted into plasma, hepcidin binds to ferroportin (FPN) in different cells, such as enterocytes and macrophages [2]. The binding of hepcidin to FPN induces degradation of FPN in lysosomes, thus reducing its expression in the membrane of enterocytes and macrophages [2, 3]. Since FPN is the main exporter of iron out of cells, this action will sequester iron into cells and prevent iron release from enterocytes and macrophages. In this way high levels of hepcidin will reduce iron transport from cells into plasma and vice-versa [4].

Hepcidin is affected by many different factors through complex biochemical relationships. Factors that suppress hepcidin expression include: low tissue oxygen, erythropoietin, erythroferrone, vitamin D [5, 6]. On the other hand, it has been shown that iron-load, inflammation, chronic renal failure, infection upregulate hepcidin expression [7]. Hepcidin has also been studied in conditions marked with insulin resistance (IR), like diabetes mellitus type 2 (DM type 2). Hepcidin levels were shown to be increased in persons with features of metabolic syndrome [8], but also in persons with DM type 2 with high ferritin and interleukin 6 (IL6) levels [9]. In persons with DM type 2 and persons with polycystic ovary syndrome matched by weight, hepcidin and hepcidin to ferritin ratio levels are decreased [10]. More importantly, this study showed that deficient levels of hepcidin were related to parameters of IR. Interestingly, deficient hepcidin production was not observed in DM type 1, which implies that IR rather than insulin deficiency is the pathophysiological process behind hepcidin disturbance. Furthermore, in studies with streptozotocin-induced diabetes in rats, disturbance of insulin signaling was associated with low levels of hepcidin, whereas insulin treatment reversed these changes [11]. This is an important observation since iron-load is a risk factor for diabetes, while the use of phlebotomy and iron-chelation were shown to reduce this risk [12]. Considering the impracticality of regular phlebotomies and poor compliance observed with chelation therapy [13], better options are needed to reduce iron-load and consequent risk of diabetes.

Recently, other biochemical parameters have been studied in states characterized with IR. YKL-40 is one of the most interesting targets in this respect. This protein which is also known as human cartilage glycoprotein-39, is expressed in macrophages, neutrophils, chondrocytes, synoviocytes, vascular smooth cells, malignant cells [14]. Studies have shown that YKL-40 is upregulated in conditions characterized with chronic inflammation like osteoarthritis [15], rheumatoid arthritis [16], liver fibrosis [17]. YKL-40 is also upregulated in acute infections [18] (probably through mechanisms including IL6), and cancer [19].

Role of YKL-40 has been studied in diabetes mellitus as well. Levels of YKL-40 are higher in DM type 2 patients compared to non-diabetic patients [14]. This association is independent of obesity, but is related to parameters of IR and IL6, which is a marker of inflammation. It has to be mentioned that other studies have shown that obesity is related to YKL-40 levels in prebupertal children and in non-diabetic relatives of DM type 2 patients [20, 21]. It could be that the discrepancies concerning the links between obesity and YKL-40 are related to the presence or lack of glucose metabolism disturbances in these patients [14]. Another contradiction arises from observations seen in non-diabetic relatives of diabetic patients. In these persons, YKL-40 is not related to markers of IR, but is related to low levels of inflammation caused by obesity [21]. Role of YKL-40 as a marker of low grade inflammation, independent of diabetes presence, has been observed in the elderly population as well [22]. Also, studies suggest that YKL-40 cannot be used as a predictive biomarker in early gestational diabetes [23], casting a doubt on its role in the emergence of diabetes mellitus.

More consistently YKL-40 levels are upregulated in complications of DM type 2, such as albuminuria [24] and peripheral artery disease [25]. In morbidly obese persons, YKL-40 levels are in correlation with monocyte chemoattractant protein 1 (MCP1) [26]. MCP1 is a chemokine involved in recruitment of different leukocytes during inflammation [27]. It is interesting to note that YKL-40 is produced through local activation of specific cells, which means that eliminating local factors that induce YKL-40 upregulation can potentially control the rise in YKL-40 levels. As we can see studies have unveiled a complex role of YKL-40 in DM type 2, which has not been elucidated completely, and which is accompanied with contradictory observations that need to be clarified.

In order to further understand the role of hepcidin and YKL-40 in DM type 2, we sought to investigate how do hepcidin and YKL-40 levels change when patients are matched by Body Mass Index (BMI), Waist to Height Ratio (WtHR), C Reactive Protein (CRP) and creatinine levels. Also, we wanted to explore if these changes happen in the prediabetic stage. Finally, we wanted to examine how does insulin treatment affects hepcidin and YKL-40 levels in persons with DM type 2.

## Methods

### Participants

In total, we examined 80 persons, 20 were included in the control group (13 men and 7 women), 30 were included in the the group with prediabetes (19 men and 11 women), and 30 were in the group with DM type 2 on insulin therapy (18 men and 12 women). Patients were enlisted from Ohrid Regional Hospital and came from different parts of Macedonia. Participants in the group with prediabetes and DM type 2 were diagnosed according to American Diabetes Association-ADA criteria [28]. Inclusion criteria were age > 18, overweight status, no significant changes in CRP and creatinine levels. Exclusion criteria included participants with documented hemochromatosis, persons on erythropoietin therapy, persons on iron therapy and persons with renal disease. All the participants were initially informed about the purpose of the study and were included only after willingly signing the letter of consent. The work was approved by the institutional committee for evaluation of ethics of the study (approval number:1278, reference number:3424) in respect of principles of the Declaration of Helsinki.

### Methods

Blood samples were taken after overnight fasting using two types of containers, one of which contained ethylenediamine tetra-acetic acid, while the other did not contain any anticoagulant. Plasma was frozen and stored at −80 °C until assayed. Complete blood count and hemoglobin were analysed by automatic cell counter (ERMA 750 Hematology Analyzer, Erma Inc., Japan), whereas serum samples were analysed by automatic biochemical analyzer (Miura Biochemical Analyzer, I.S.E. SRL, Italy). Serum ferritin, hepcidin and YKL-40 were measured by ELISA method with ELISA kits used according to manufacturers instructions (Elabscience Biotechnology Co. Ltd). The kits contain micro ELISA plates precoated with specific antibodies. Serum samples were added to ELISA plate wells. The minimum detectable dose of human ferritin was 9.375 ng/ml, with a detection range of 15.625-1000 ng/ml and coefficient of variation < 10%. For hepcidin the minimum detectable dose was 0.094 ng/ml, with a detection range of 0.156-10 ng/ml and coefficient of variation < 10%. For YKL-40 the minimum detectable dose was 37.5 pg/ml with a detection range of 62.5-4000 pg/ml and coefficient of variation < 10%.

### Statistical analysis

Statistical analysis was done using SPSS 21, IBM software. Analysis between groups for mean comparisons was done using One Way ANOVA, after initially testing for distribution of normality using Kolomogorov-Smirnov and Shapiro-Wilk test. Skewed data were transformed using log_10_. When the distribution of data did not meet the criteria for parametric tests, we used the Kruskal Wallis test. Correlation was done using Spearman test. Results are presented as mean ± SD, median (interquartile range) and geometric mean with 95% confidence intervals (CIs). In all cases, *p* < 0,05 was considered statistically significant.

## Results

**Fig. 1:**
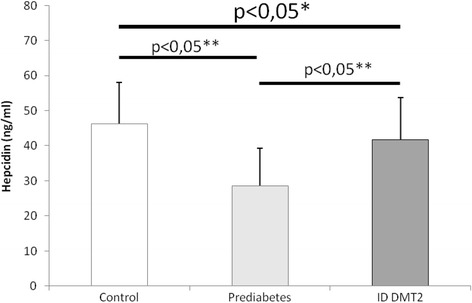
Hepcidin levels in control, prediabetic patients and diabetic patients dependent on insulin. There is a statistical difference between all groups. Levels of hepcidin go down in prediabetes, but recuperate in insulin dependent DM type 2. *Statistical analysis done using One-Way ANOVA. **Post-hoc analysis of differences between specific groups. Abbreviations. ID DMT2, insulin dependent diabetes mellitus type 2

**Fig. 2:**
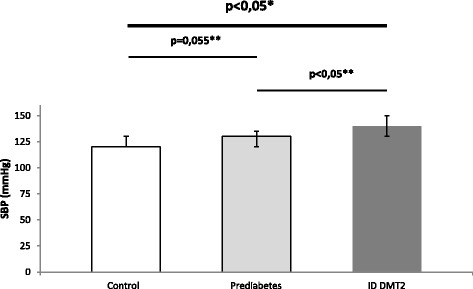
Systolic blood pressure levels in control, prediabetic patients and diabetic patients dependent on insulin. Statistical differences between all groups are significant. Median systolic blood pressure levels rise nearly significantly in prediabetes compared to control. The rise in median systolic blood pressure is significant in diabetic patients compared to prediabetic patients. *Statistical analysis done using One-Way ANOVA. **Post-hoc analysis of differences between specific groups. Abbreviations. ID DMT2, insulin dependent diabetes mellitus type 2

**Fig. 3:**
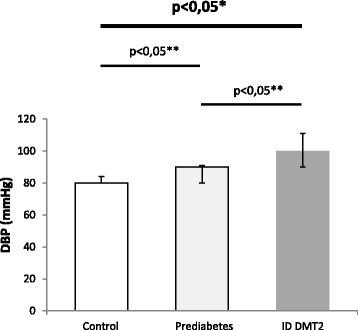
Diastolic blood pressure levels in control, prediabetic patients and diabetic patients dependent on insulin. There is a statistically significant difference between all groups. Median levels of diastolic blood pressure rise in prediabetes compared to control and diabetes compared to prediabetes. *Statistical analysis done using One-Way ANOVA. **Post-hoc analysis of differences between specific groups. Abbreviations. ID DMT2, insulin dependent diabetes mellitus type 2

**Fig. 4:**
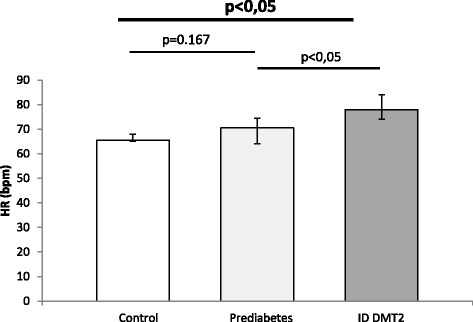
Heart rate levels in control, prediabetic patients and diabetic patients dependent on insulin. Heart rate increase in all groups is statistically significant. Specific between group analysis reveals increase in median levels in prediabetes compared to control, but this is not significant. On the other hand, median levels rise in diabetes compared to prediabetes with statistical significance. *Statistical analysis done using One-Way ANOVA. **Post-hoc analysis of differences between specific groups. Abbreviations. ID DMT2, insulin dependent diabetes mellitus type 2

Median age was 48 (39-58.25) for the control group, 53.5 (37.5-59.25) for prediabetic patients and 59,5 (53.75-70.25) for diabetic patients. Hepcidin levels differed significantly between all groups (*p* = 0,00001). Post-hoc analysis showed significant differences between control and prediabetic patients (*p* < 0.05) but also between prediabetic and diabetic patients on insulin therapy (*p* < 0.05) (Fig. 1). Systolic blood pressure (SBP) values showed significant differences between all groups (*p* < 0.0001). Post-hoc analysis revealed nearly significant differences between control and prediabetic patients (*p* = 0.055) while the difference between prediabetic patients and diabetic patients on insulin was statistically significant (*p* < 0.05) (Fig. 2). Diastolic blood pressure (DBP) showed significant differences between all groups (*p* < 0.0001). Post-hoc analysis revealed significant differences between control and prediabetic patients, but also between prediabetic patients and diabetic patients on insulin therapy (*p* < 0.05) (Fig. 3). YKL-40 levels did not differ significantly between groups (*p* = 0.294). Heart rate (HR) also differed significantly between all groups (*p* < 0.0001), which was confirmed by post-hoc analysis between control and prediabetic patients, but also between prediabetic patients and diabetic patients on insulin therapy (Fig. 4). Summarized clinical, biochemical and anthropometrical data are presented in Table 1.

**Table 1:** Clinical, anthropometrical and biochemical data by groups

Parameters	Control group (*n* = 20)	Persons with prediabetes (*n* = 30)	Persons with type 2 diabetes on insulin therapy (*n* = 30)
BMI (kg/m^2^)	27.9 ± 3.4	27.4 ± 4.5	27.2 ± 2.5
WHtR	0.59 ± 0.08	0.59 ± 0.11	0.59 ± 0.10
CRP (mg/l)	0.0(0.0-0.0)	0.0(0.0-0.0)	0.0(0.0-0.25)
Creatinine (μmol/l)	76.3 ± 5.1	80.9 ± 11.7	80.8 ± 8.5
Urea (mmol/l)	5.2(4.3-6.3)	4.7(4.0-5.9)	5.1(4.3-6.0)
Glucose (mmol/l)	4.9 ± 0.4	6.0 ± 0.4	9.5 ± 2.6
HbA1C (%)	4.1 ± 0.5	6.2 ± 0.1	9.1 ± 2.1
Serum iron (μmol/l)	16.6 ± 5.4	19.4 ± 5.9	18 ± 6.1
TIBC (μmol/l)	49.3 ± 4.6	51.0 ± 7.6	51 ± 7.1
UIBC (μmol/l)	32.7 ± 3.0	31.7 ± 3.1	33.0 ± 2.4
TS (%)	33.1 ± 8.8	37.2 ± 6.8	34.4 ± 8.4
Hgb (g/dl)	14.2 ± 1.4	14.4 ± 1.5	13.9 ± 1.4
**Hepcidin (ng/ml)**	**46.3 ± 11.8**	**28.6 ± 10.8**	**41.8 ± 11.9**
Ferritin (ng/ml)	130.5(39.9-156.7)	152.5(104.1-162.4)	143.9(91.1-155.8)
YKL-40 (ng/ml)	75.1(55.5-146.1)	70.8(43.8-127.0)	58.4(44.3-82.2)
**HR (bpm)**	**65.5(65-68)**	**70.5(64-74.5)**	**78(74-84)**
**SBP (mmHg)**	**120(120-130)**	**130(120-135)**	**140(130-150)**
**DBP (mmHg)**	**80(80-84)**	**90(80-91)**	**100(90-111)**

*BMI* body mass index, *WHtR* waist to height ratio, *CRP-C* reactive protein, *HbA1C* glycated hemoglobin, *TIBC* total iron binding capacity, *UIBC* unsaturated iron binding capacity, *T* transferrin saturation, *Hgb* hemoglobin, *HR* heart rate, *SBP* systolic blood pressure, *DBP* diastolic blood pressure. Results are expressed as mean ± SD and median (Q1-Q3). Variables with significant differences between groups are shown in bold (*p* < 0.05)

**Fig. 5:**
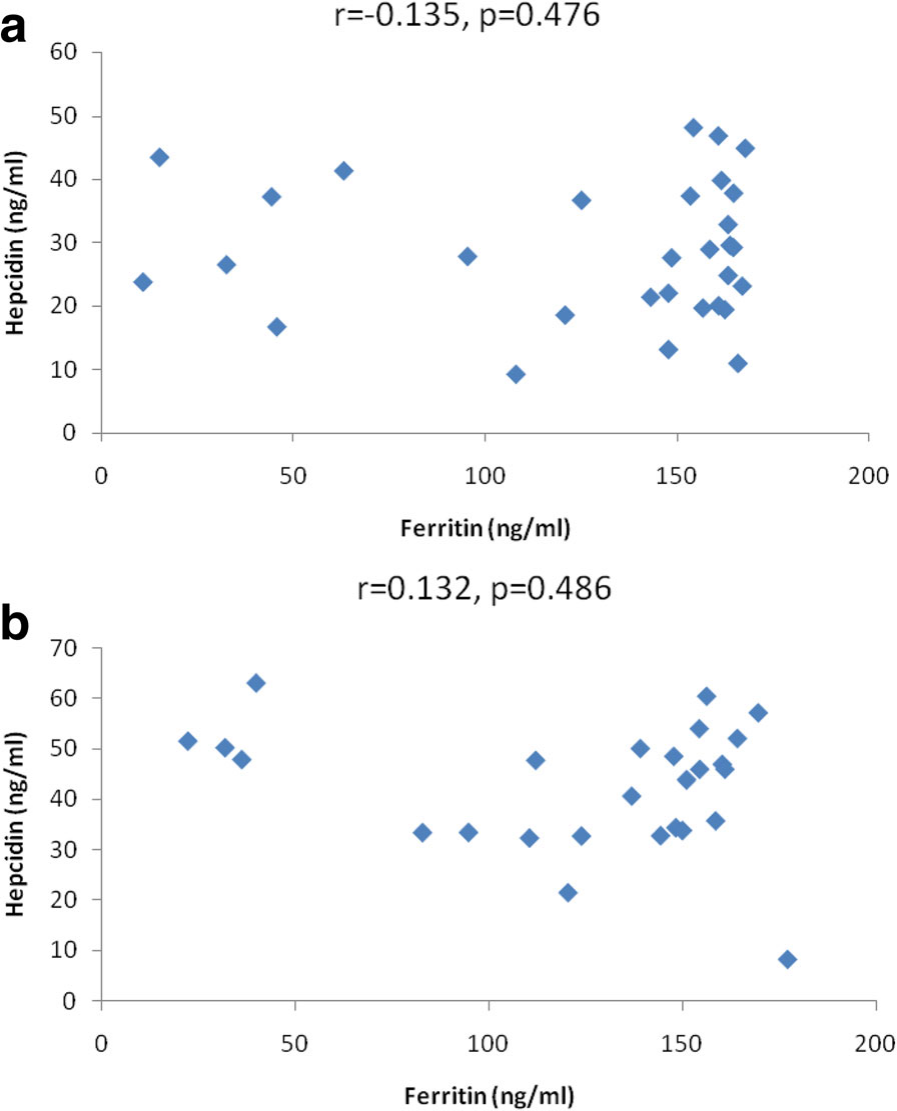
Correlations between hepcidin and ferritin in prediabetic patients and diabetic patients dependent on insulin. Spearman correlation revealed no significant correlation between hepcidin and ferritin in (**a**) prediabetes and (**b**) diabetes

**Fig. 6:**
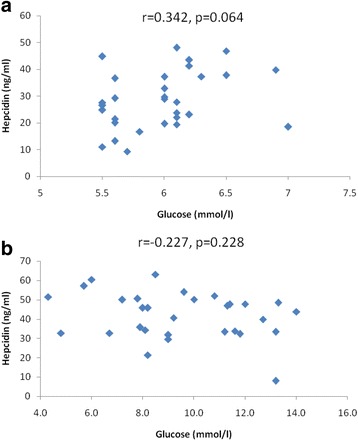
Correlations between hepcidin and glucose in prediabetic patients and diabetic patients dependent on insulin. Spearman correlation revealed no significant correlation between hepcidin and glucose in (**a**) prediabetes and (**b**) diabetes

**Fig. 7:**
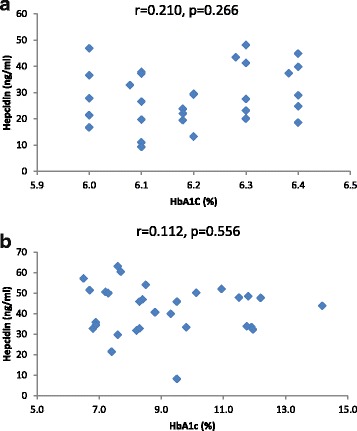
Correlations between hepcidin and HbA1C in prediabetic patients and diabetic patients dependent on insulin. Spearman correlation revealed no significant correlation between hepcidin and HbA1C in (**a**) prediabetes and (**b**) diabetes

**Fig. 8:**
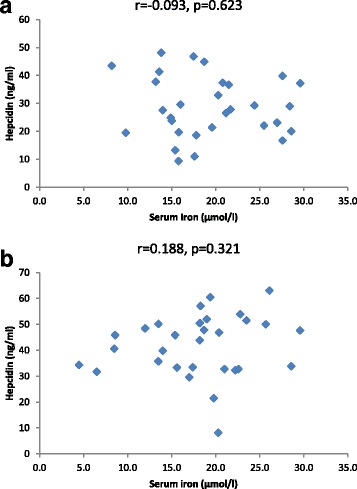
Correlations between hepcidin and serum iron in prediabetic patients and diabetic patients dependent on insulin. Spearman correlation revealed no significant correlation between hepcidin and serum iron in (**a**) prediabetes and (**b**) diabetes

Hepcidin levels in all groups did not correlate with ferritin (Fig. 5), glucose (Fig. 6), glycated hemoglobin (HbA1C) (Fig. 7) and iron levels (Fig. 8). Although hepcidin was positively correlated with glucose in persons with prediabetes, this was not significant (*p* = 0.064). Summary of the most important correlation coefficients are presented in Table 2.

**Table 2:** Selected Spearman correlations between variables in all three groups

Correlations	Control group (*n* = 20)	Persons with prediabetes (*n* = 30)	Persons with type 2 diabetes on insulin therapy (*n* = 30)
YKL-40/age	*r* = 0.243, *p* = 0.302	*r* = −0.073, *p* = 0.703	*r* = 0.046, *p* = 0.809
Hepcidin/Glucose	*r* = 0.150, *p* = 0.528	*r* = 0.342, *p* = 0.064	*r* = −0.227, *p* = 0.228
Hepcidin/HbA1C	*r* = −0.154, *p* = 0.517	*r* = 0.210, *p* = 0.266	*r* = 0.112, *p* = 0.556
Hepcidin/Ferritin	*r* = −0.081, *p* = 0.734	*r* = 0.082, *p* = 0.666	*r* = 0.132, *p* = 0.486
Hepcidin/YKL-40	*r* = 0.188, *p* = 0.427	*r* = −0.034, *p* = 0.858	*r* = −0.358, *p* = 0.052
Ferritin/YKL-40	*r* = −0.173, *p* = 0.466	*r* = −0.135, *p* = 0.476	*r* = −0.009, *p* = 0.964
**BMI/WHtR**	***r*** **= 0.460,** ***p*** **= 0.041**	***r*** **= 0.828,** ***p*** **< 0.001**	***r*** **= 0.459,** ***p*** **= 0.011**
**HR/SBP**	***r*** **= 0.800,** ***p*** **< 0.001**	***r*** **= 0.767,** ***p*** **< 0.001**	***r*** **= 0.630,** ***p*** **< 0.001**
**HR/DBP**	***r*** **= 0.531,** ***p*** **= 0.016**	***r*** **= 0.873,** ***p*** **< 0.001**	***r*** **= 0.657,** ***p*** **< 0.001**

Significant correlations are shown in bold (*p* < 0.05)

## Discussion

Studies in humans and rats have shown that IR affects hepcidin levels [10, 29]. In our study, levels of hepcidin were lower in persons with prediabetes compared to controls, when matched for BMI, WtHR, creatinine and CRP values. Other studies have shown similar changes in persons with diabetes when matched for BMI and CRP levels [10]. However, our study, for the first time to our knowledge, revealed that changes in hepcidin levels start to happen earlier, that is, in the prediabetic stage. Prediabetes is a risk factor for diabetes and it is a known condition associated with IR. Also, studies that have shown increased levels of hepcidin in diabetes did not have persons matched for factors that influence hepcidin production. In these studies persons with diabetes either had high BMI [30], renal disease [31], or different features of metabolic syndrome compared to controls. Since inflammation upregulates hepcidin expression, our participants were also matched by inflammatory status by not having significant changes in CRP values. Our results from patients with prediabetes are in terms with studies that confirm the predictive value of hepcidin in incident DM type 2; lower levels of hepcidin are predictors of incident DM type 2, while higher levels of hepcidin are related with a decreased risk for DM type 2 [32].

In addition, the present study, for the first time to our knowledge, shows that persons with diabetes on insulin therapy have higher values of hepcidin, confirming similar results obtained from studies on rats [11]. Different studies have shown that hepcidin levels in DM type 2 are low [10, 33]. Since insulin therapy in models of rats does recuperate hepcidin expression we believe that this is the case with our patients as well. By restoring hepcidin levels we can control iron-load in cells and potentially prevent DM type 2 [34]. But maintaining adequate hepcidin levels can be beneficial for patients with overt DM type 2 as well. The reason for this is that complications of diabetes can be prevented by reducing iron-load [34].

We can speculate as to why hepcidin levels go down in prediabetes. It could be that some sort of a feedback loop is activated in such a way that supranormal levels of insulin cause compensatory downregulation of hepcidin (as it happens in prediabetes), while in states with subnormal levels of hepcidin (like full blown diabetes) treatment with insulin restores insulin signaling and corrects hepcidin levels. Another theory could be that changes in hepcidin levels might occur as a result of disturbances in secretion of hepcidin by beta cells of pancreas. We know that hepcidin is expressed in beta cells of pancreas, and more importantly in secretory granules containing insulin [35]. Since many products of endocrine pancreatic cells pass into plasma, it may be that, serum hepcidin can be affected in states characterized with glucose metabolism impairment. Future experimental studies should clarify the role of liver and local pancreatic secretion of hepcidin in the patophysiology of diabetes.

The results of this study showed that when eliminating factors that influence the production of YKL-40, serum levels of this protein do not change significantly in persons with prediabetes and diabetes. Studies examining YKL-40 levels in diabetes and prediabetes have shown that the rise in serum levels of YKL-40 is an indirect response to local inflammatory status, morbid obesity, renal dysfunction [24–26]. Our study is the first one to study changes in serum YKL-40 levels when corrected for known upregulating factors. Since YKL-40 levels did not change in our setting, we can say that the role of YKL-40 in the patophysiology of diabetes is more secondary compared to hepcidin disturbance.

Additionally, our results have also shown that patients with prediabetes and DM type 2 have signs of autonomic dysfunction. This was confirmed by significant increase in median values for HR, SBP and DBP across the groups. This is in line with previous reports concerning similar changes observed in diabetic patients [36, 37].

## Conclusions

According to results of our study, we conclude that prediabetes is connected with low values of hepcidin when patients are matched for other factors, whereas treatment with insulin corrects hepcidin values in persons with diabetes. Our diabetic patients had uncontrolled diabetes, which was confirmed by high levels of mean glucose and HbA1C. This would preclude metabolic regulation as the “culprit” behind increased levels of hepcidin in diabetic patients. This study shows that insulin therapy can have beneficial effects outside its classical actions on glucose metabolism. The new paradigm connects insulin therapy with the potential to correct frequently observed changes of iron metabolism in diabetes [34]. This action of insulin is attributed to its effects in restoring hepcidin levels. Yet, we still do not know what is the role of insulin dosage, patient compliance and duration of insulin therapy in relation to hepcidin levels. Also, long-term measurements of hepcidin levels should be evaluated to better understand the role of hepcidin fluctuations in DM type 2. Future studies should resolve these important questions.

## Abbreviations

BMI: Body mass index
CRP: C Reactive protein
DBP: Diastolic blood pressure
DM type 2: Diabetes mellitus type 2
FPN: Ferroportin
HbA1C: Glycated hemoglobin
HR: Heart rate
IL6: Interleukin 6
IR: Insulin resistance
MCP1: Monocyte chemoattractant protein 1
SBP: Systolic blood pressure
WtHR: Waist to height ratio
